# Progressive Symptoms in an Older Patient Recently Diagnosed With Coeliac Disease and Rapid Progression to Enteropathy-Associated T-cell Lymphoma (EATL)

**DOI:** 10.7759/cureus.74215

**Published:** 2024-11-22

**Authors:** Eslam G Muhammed, Michael Davies, Asim Hamza, Michael Wall

**Affiliations:** 1 Gastroenterology and Hepatology, Blackpool Victoria Hospital, Liverpool, GBR; 2 Gastroenterology and Hepatology, Countess of Chester Hospital NHS Trust, Chester, GBR; 3 Gastroenterology, Countess of Chester Hospital NHS Trust, Chester, GBR; 4 Histopathology, Countess of Chester Hospital NHS Trust, Chester, GBR

**Keywords:** coeliac disease, enteropathy-associated t-cell lymphoma (eatl), old patients, rapid progression lymphoma, refractory coeliac disease

## Abstract

Coeliac disease (CD) is an immune-mediated condition that causes damage to the small intestine upon gluten consumption by genetically susceptible individuals. To determine whether there is an active coeliac disease or the presence of additional pathologies, patients must undergo regular evaluations, including repeat endoscopy.

In this analysis, we present a case study of a 75-year-old woman from England who was diagnosed with coeliac disease later in life. She carries the HLA-DQ2 genetic marker and developed type 2 enteropathy-associated T-cell lymphoma (EATL) 12 months after her diagnosis of coeliac disease.

## Introduction

Diagnosing coeliac disease in older adults can be quite challenging for primary care providers. These difficulties may arise from subtle clinical symptoms, a general lack of awareness about coeliac disease in this age group, and a tendency to prioritize the diagnosis of more severe conditions such as cancers [1,2]. For instance, subtle changes in gastrointestinal patterns may be linked to physiological modifications in the intestinal pathway caused by conditions such as irritable bowel syndrome, affective disorders (including anxiety and depression) or even be considered a part of the normal ageing process.

Once a diagnosis of coeliac disease is confirmed, patients typically experience both clinical improvement and mucosal healing within 12 months by eliminating gluten through a gluten-free diet. However, some individuals with coeliac disease may show a delayed response to this strict diet and continue to experience symptoms and incomplete recovery beyond the initial 12 months. For these individuals, complete recovery may take 18 to 24 months [3]. In such cases, the recommended approach is to closely monitor the patient and adopt a 'wait-and-see' strategy to avoid unnecessary invasive procedures and potentially harmful treatments such as immunosuppressive medications and steroids. While many patients manage coeliac disease effectively through diet, there is a rare but serious risk of complications, such as enteropathy-associated T-cell lymphoma (EATL), especially in older adults. EATL is a rare form of lymphoma that can arise in long-standing or untreated coeliac disease [4].

## Case presentation

A 76-year-old female patient presented to her GP with persistent diarrhoea and a weight loss of 19 kg over the past 16 months. Apart from a previous stroke with no residual symptoms, there was no significant past medical history. The coeliac screening was conducted as part of the initial blood tests, which returned positive: anti-tissue transglutaminase antibodies (tTG) 18.0 U/mL and anti-endomysial antibodies (EMA) positive. The patient subsequently underwent a gastroscopy, which endoscopically showed scalloping within D2 and histological features in keeping with coeliac disease (subtotal villous atrophy, increase in intraepithelial lymphocytes and crypt hyperplasia. The patient was not known to have coeliac disease previously and had not suffered from GI symptoms, such as diarrhoea, when consuming gluten throughout her life.

**Figure 1:**
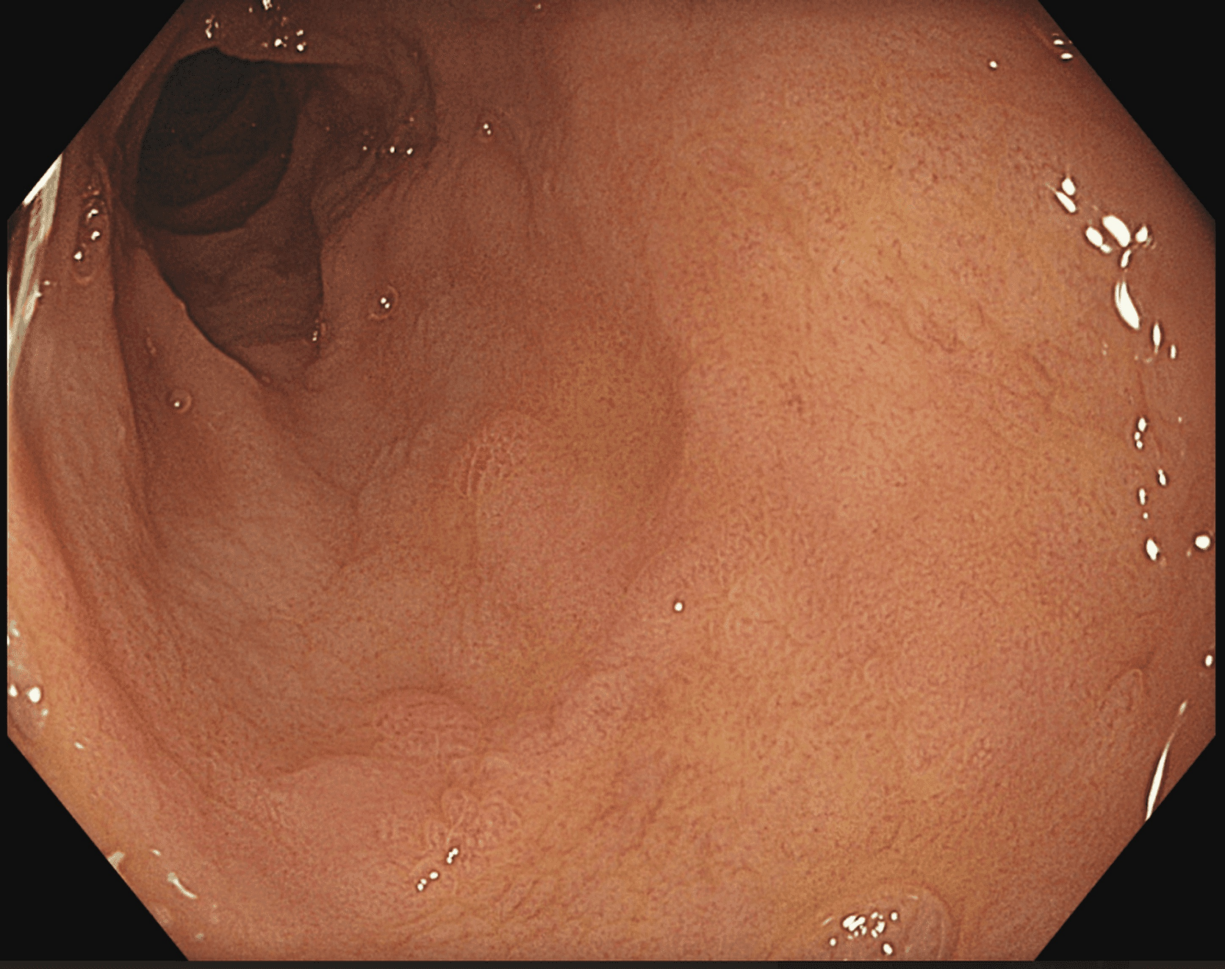
Endoscopic picture showing duodenal atrophy This indicates a non-response to a gluten-free diet.

**Figure 2:**
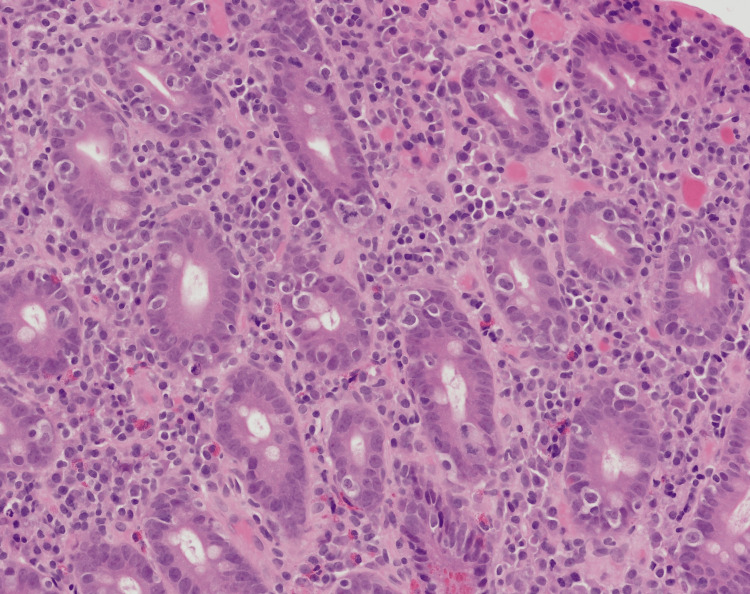
The slide shows scattered, enlarged, irregular and hyperchromatic lymphocytes within the crypt epithelium. Some of the lymphocytes demonstrated prominent nucleoli and a tripolar mitotic figure was identified, which indicates a high likelihood of developing malignancy.

Despite strict adherence to a gluten-free diet (GFD), the patient had ongoing diarrhoea and weight loss. The Dietitian team reviewed the patient to ensure dietary adherence and no inadvertent exposure to gluten. Symptoms continued, and TTG and EMA serology remained positive, so repeat gastroscopy was performed 13 months after the initial diagnosis. Endoscopically, the duodenum appeared atrophic (Figure 1), and duodenal biopsies demonstrated total villous atrophy and increased intraepithelial lymphocytes. Along with this, atypical histological changes were observed, consistent with rapidly progressive celiac disease to type 2 refractory coeliac disease (RCD), with early progression to enteropathy-associated T-cell lymphoma (EATL) (Figure 2). The histological findings from the initial gastroscopy were reviewed, showing no evidence of changes suggestive of RCD.

**Figure 3:**
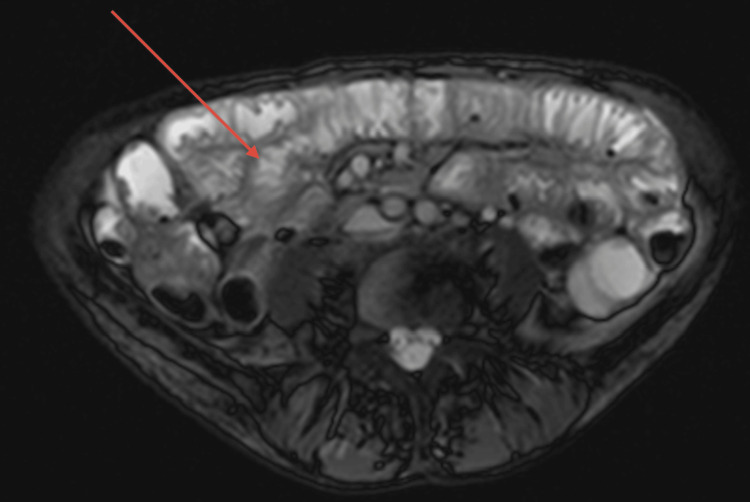
MRI small bowel showing thickening of the folds and jejunization of the ileal loops, which indicates a high likelihood of developing malignancy.

**Figure 4:**
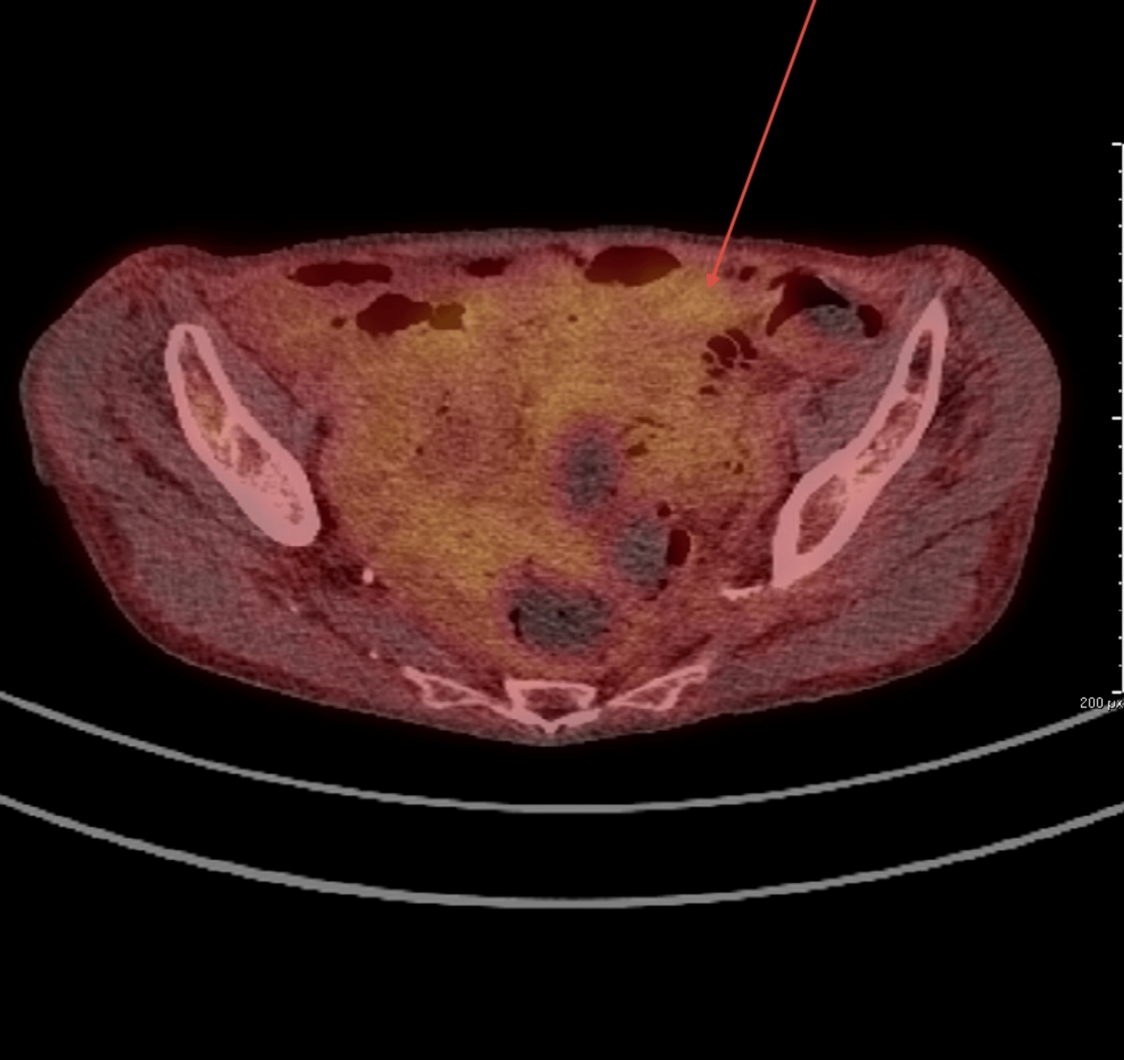
CT PET showing low-grade uptake in the bowel, with multiple, small, poorly avid mesenteric lymph nodes This indicates a high likelihood of developing malignancy. PET: positron emission tomography

An MRI of the small bowel was performed, showing thickening of the folds and jejunization of the ileal loops consistent with active coeliac disease (Figure 3). The CT PET scan was also conducted., showing low-grade uptake in the bowel, with multiple small, poorly avid mesenteric lymph nodes (Figure 4).

The patient was commenced on budesonide and demonstrated a dramatic improvement in clinical symptoms, with diarrhoea fully resolving within 48 hours and an improving appetite. The patient was referred to the regional RCD centre and locally to Haematology, subsequently commencing chemotherapy for EATL. The patient remains clinically well with no GI symptoms and improved weight (13 kg in 6 months).

## Discussion

Coeliac disease can present similarly with other causes of diarrhoea such as small intestinal bacterial overgrowth (SIBO), irritable bowel syndrome (IBS), and microscopic colitis. While the most common cause for ongoing villous atrophy at repeat endoscopy is inadvertent gluten exposure (30-50%), careful clinical and dietetic review is required to assess if this is the case; the patient may be a slow responder to a GFD or if refractory coeliac disease (RCD) should be considered. While refractory celiac disease (RCD) is rare, seen in only around 1% of patients with CD, it poses a risk for malignancy, including enteropathy-associated T-cell lymphoma (EATL) [5].

While the majority are diagnosed in childhood/early adulthood, one-fifth of patients are diagnosed over the age of 65. A GFD diet is the mainstay of treatment for both clinical and endoscopic remission in most patients. However, a notable proportion of patients (up to 25%) will have ongoing symptoms such as diarrhoea and weight loss despite a GFD [6], in other terms, non-responsive coeliac disease (NRCD). This is complicated by the fact that monitoring anti-tissue transglutaminase antibodies and symptoms correlate poorly with the presence of active coeliac disease and ongoing inflammation. Here, it is essential that patients are reviewed to assess potential causes of their NRCD. Whilst the most common cause (30-50%) is gluten exposure (inadvertent or non-compliance) [7-9], there are multiple other potential causes for ongoing symptoms that require careful clinical and dietetic review. These include slow response to a GFD, gluten hypersensitivity, co-existing pathologies such as microscopic colitis or irritable bowel syndrome, or rarely, RCD [10].

RCD, fortunately, is uncommon, being seen in only around 1% of patients with CD. RCD has two distinct forms: Type 1 is characterized by normal intraepithelial lymphocytes and Type 2 is characterized by an abnormal T-cell population and the risk of malignant transformation to EATL [11]. EATL is more common in patients of advanced age and those presenting/ diagnosed at an older age, and both features of our patient’s presentation. Budesonide, as in our patient’s case, has been shown to be effective in the initial treatment for RCD (both Types 1 and 2). However, long-term treatment of Type 2 RCD often involves chemotherapeutic agents.

## Conclusions

We present a case of rapid progression from the initial diagnosis of coeliac disease to RCD, highlighting the importance of monitoring older patients newly diagnosed with CD, as they are at a higher risk of potential for rapid progression to EATL. To ensure a regular review of symptoms and progress, with a low threshold for repeat assessment if there is failure to respond to a GFD, given the increased risk of complications such as RCD and EATL.
